# Urban Adolescence: The Role of Neighbourhood Greenspace in Mental Well-Being

**DOI:** 10.3389/fpsyg.2021.712065

**Published:** 2021-09-16

**Authors:** Marie A. E. Mueller, Eirini Flouri

**Affiliations:** Department of Psychology and Human Development, Institute of Education, University College London, London, United Kingdom

**Keywords:** greenspace, neighbourhood, adolescence, well-being, garden, safety, physical activity

## Abstract

Mental health and well-being in adolescence are associated with many short- and long-term outcomes. The evidence suggests that greenspace may play a role in adolescents' mental well-being, but we do not know much about the specifics of this link. In this paper, we investigated the role of other factors in the association. In a cross-sectional study, we investigated the role of neighbourhood greenspace in emotional and behavioural outcomes in 11-year-old urban adolescents participating in the UK Millennium Cohort Study (*n* = 4,534). We used linear regression models to test for an association of greenspace with self-esteem, happiness, positive mood, negative mood, and antisocial behaviour. We also investigated effect modification/moderation by garden access, physical activity, and perceived area safety. We did not find a main effect of greenspace, but we did find interaction effects. First, in adolescents without a garden, higher levels of greenspace were associated with lower levels of self-esteem and positive mood. Second, in adolescents who reported lower levels of physical activity, higher levels of greenspace were associated with lower levels of negative mood. Third, in adolescents who perceived their areas to be unsafe, higher levels of greenspace were associated with higher levels of antisocial behaviour. Our findings suggest that merely more greenspace in the neighbourhood may not be sufficient to promote the mental well-being of urban adolescents in the UK. However, greenspace does seem to have an influence under certain conditions which should be investigated further in future studies.

## Introduction

A large body of evidence suggests a relationship between exposure to nature, particularly greenspace, and physical and mental health in both adults and children (Bowler et al., 2010; Hartig et al., 2014; Twohig-Bennett and Jones, 2018; Vanaken and Danckaerts, 2018). Greenspace has many positive functions that are likely to facilitate healthy child development. These include promoting recovery from stress, encouraging engagement in physical and social activities, and reducing exposure to environmental stressors (Markevych et al., 2017). Indeed, the evidence to date suggests a positive link between exposure to greenspace and several child and adolescent outcomes, including emotional and behavioural adjustment (Tillmann et al., 2018; Weeland et al., 2019b). It remains unclear, however, whether other factors may modify or moderate this link. Some studies indicate that children are not equally affected by greenspace, but that the link is, indeed, modified or moderated by other factors, such as sex, socio-economic status, and ethnicity (Taylor et al., 2002; Balseviciene et al., 2014; Flouri et al., 2014; McEachan et al., 2018). In the present study, in which we focus on early adolescence, we take this complexity into account by looking at a range of emotional and behavioural outcomes and by investigating the role of other factors that may modify or moderate the association.

### Greenspace and Adolescent Mental Health and Well-Being

Mental health and well-being in adolescence are the foundation for a healthy life. Mental health problems, in turn, have been linked to many adverse outcomes, both in the short- and in the long-term, including higher distress, lower academic outcomes, poorer social relationships, and poorer employment prospects in adulthood (Kim-Cohen et al., 2003; Goodman et al., 2011; Department of Health, 2013; Heizomi et al., 2015; Ford and Parker, 2016). For example, lower self-esteem in adolescence is associated with poorer physical and mental health, engagement in criminal behaviour, and poorer economic prospects in adulthood (Trzesniewski et al., 2006; Masselink et al., 2018); bullying in adolescence is associated with future antisocial, criminal, and violent behaviour (Renda et al., 2011); and depressive symptoms in adolescence are linked to an increased risk of developing major depressive disorder later in life (Fergusson et al., 2005; Johnson et al., 2009). It is important to identify both the factors that may put adolescents at risk of developing emotional and behavioural problems and the factors that may protect and promote their mental health and well-being. The evidence suggests a role of several proximal factors, such as sex, maltreatment, parenting, family structure, and parental mental health (Brown et al., 1999; Griffin et al., 2000; Brennan et al., 2002; Demuth and Brown, 2004; Parker and Benson, 2004; Card et al., 2008; Mersky et al., 2012), but research has also highlighted the role of distal factors, including factors of the residential neighbourhood. Parents' perception of the quality of their neighbourhood is associated with externalising problems and depression in adolescents (Eamon, 2001; Ford and Rechel, 2012; Li et al., 2017); adolescents' perception of neighbourhood cohesion is linked to their emotional well-being (Aminzadeh et al., 2013); and adolescents' fear of crime is associated with emotional and behavioural difficulties (Mueller et al., 2019). The role of nature, particularly greenspace, as a protective or promotive factor of emotional and behavioural adjustment has received increasing attention in recent years.

Indeed, several studies do suggest an association of greenspace with children's and adolescents' emotional and behavioural adjustment (Vanaken and Danckaerts, 2018; Weeland et al., 2019b). For example, Feda et al. (2015) found that higher levels of residential park area were associated with lower levels of perceived stress in 68 12- to 15-year-old adolescents from Buffalo, New York. Further, Li et al. (2018) tracked the movements of 155 adolescents at the age of 13–19 years from Illinois, over a period of 4 days, and found that greater exposure to nature was associated with lower scores on depression, anger, and fatigue, and with better overall mood. Finally, Younan et al. (2016) studied the link between greenspace and aggressive behaviour in 1,287 adolescents from California (followed from 9 to 18 years of age) and found a negative relationship between greenspace and aggression.

This was only a selection of studies to describe some of the evidence that does suggest a relationship between greenspace and adolescent mental health and well-being. It is important to note, however, that some studies did not find a link and that many studies find a link only for a specific subset or combination of exposures and outcomes. Therefore, although the link is certainly plausible, the evidence to date is inconclusive (for reviews, see Vanaken and Danckaerts, 2018; Weeland et al., 2019b; Zhang et al., 2020).

### The Potential Role of Other Factors

It is likely that the association of greenspace with adolescent mental health and well-being is modified or moderated by other factors. In our study, we explored the effects of three potential, but understudied, moderators and modifiers: perceived area safety, physical activity, and access to a garden. Do these factors play a role in the link between neighbourhood greenspace and adolescent mental health and well-being?

Adolescents' perceptions of the safety of their neighbourhoods may be an indicator (a) of the quality of greenspaces and (b) of how often adolescents visit them. Adolescents who perceive their neighbourhoods to be safe may visit nearby greenspaces more often than their counterparts. This is suggested by studies showing that area safety and crime are linked to young people's outdoor physical activity (Gómez et al., 2004; Molnar et al., 2004; Ries et al., 2008). The link between neighbourhood greenspace and adolescents' mental health and well-being may, therefore, be moderated by perceived area safety. A similar effect has already been shown in studies on adults that found that area safety, deprivation, and incivility affect use of greenspaces and moderate the link between greenspace and well-being (Jones et al., 2009; Chong et al., 2013).

Adolescents' levels of physical activity may also affect how much they benefit from nearby greenspaces. Physical activity is one of the main reasons for adolescents to visit greenspaces (Bloemsma et al., 2018), which suggests that more active adolescents may visit greenspaces more often than their counterparts. Although physical activity has been discussed as a potential mediator (Markevych et al., 2017), studies on adults do suggest that physical activity may also be a moderator of the link between greenspace and health (Astell-Burt et al., 2013; McEachan et al., 2016).

Finally, access to a garden may also play a modifying role. It has already been shown that garden access benefits the emotional and behavioural adjustment of children in the UK (Flouri et al., 2014; Richardson et al., 2017). However, access to a garden may also modify the link between neighbourhood greenspace and mental health and well-being. A garden offers an immediate opportunity for exposure to nature, and it is possible that adolescents who have access to a garden visit other greenspaces less often than their counterparts. Therefore, adolescents without a garden may benefit more from greenspaces in their neighbourhoods. This compensation effect of proximal greenspace on visits to other natural environments has been discussed previously (Maat and de Vries, 2006). It is also possible, however, that adolescents who have access to a garden are more connected to nature and seek other greenspaces more often than those without. This is suggested by studies on adults that found that use of gardens (for relaxation or gardening) was linked to use of parks and visits to nature (Lin et al., 2014; de Bell et al., 2020).

### The Present Study

In the present study, we investigated the association of neighbourhood greenspace with self-esteem, happiness, positive mood, negative mood, and antisocial behaviour in 11-year-old adolescents from urban areas in the UK. Our study adds to the literature in three ways. First, we used data on a large sample and included a range of emotional and behavioural outcomes, allowing us to test for an effect of greenspace by outcome domain. Second, we explored effects in early adolescence when urban children in the UK begin to move independently around their neighbourhoods and when exposure to greenspace is not entirely due to parents. Third, we explored the role of three potential moderators, thereby specifying some of the conditions under which an effect of greenspace on mental health and well-being may be accentuated or attenuated.

## Methods

### Study Sample

We used data from the UK Millennium Cohort Study (MCS; https://cls.ucl.ac.uk/cls-studies/millennium-cohort-study/). The MCS is a birth cohort study that follows 19,243 families in the UK with children born between 2000 and 2002. The MCS sample is disproportionately stratified to ensure adequate numbers for the four UK countries and for UK electoral wards with disadvantaged or ethnic minority populations (see Plewis, 2007). To this day, children have been followed across seven sweeps, from 9 months (at Sweep 1) to 17 years (at Sweep 7). At each sweep, data is collected on a wide range of outcomes and influences, including children's physical, socio-emotional, cognitive, and behavioural development; their daily life, behaviour, and experiences; and economic circumstances, parenting, relationships, and family life. We used cross-sectional data of Sweep 5 (January 2012–February 2013) when children were around 11 years old. We studied the link between greenspace quantity and self-reported self-esteem, happiness, positive mood, negative mood, and antisocial behaviour. Our analytic sample included adolescents who had lived in urban areas at Sweep 1, had never changed address until the age of 11 years (Sweep 5), and had valid data on at least one of the five outcomes (*n* = 4,534). Our non-analytic sample included the remaining MCS children (*n* = 14,709). Restricting our sample to adolescents who had never moved ensured that our measure of neighbourhood greenspace reflected exposure to greenspace not only at Sweep 5 but throughout childhood. It also kept the adolescents' neighbourhood history consistent and thereby avoided the introduction of bias by changes in type of neighbourhood due to household move. In addition, because a large majority of MCS children lives in urban areas (80% at Sweep 1) and because urbanicity modifies the link between greenspace and health (Mitchell and Popham, 2007), we excluded rural children from our sample.

### Study Variables

#### Emotional and Behavioural Outcomes

We investigated five outcomes (i.e., self-esteem, happiness, positive mood, negative mood, and antisocial behaviour), all of which were self-reported at Sweep 5. This is the first time that the cohort members completed a questionnaire in that they answered questions about their mental health and well-being that included more sensitive questions on self-esteem and antisocial behaviour. The questionnaire can be found online on the MCS website (https://cls.ucl.ac.uk/cls-studies/millennium-cohort-study/mcs-age-11-sweep/). Self-esteem was measured with five items of the validated Rosenberg self-esteem scale (Rosenberg, 1965). Happiness was measured with six items asking children about their feelings about different aspects of their lives. This scale has been used elsewhere as a measure of well-being (e.g., Bannink et al., 2016; Kelly et al., 2016, 2018). In addition to these two scales, adolescents answered questions that provided further valuable information about their well-being and behaviour. These questions asked about the children's feelings in the past 4 weeks and about their engagement in delinquent and antisocial behaviour. Because these items did not belong to a clearly defined scale, we derived underlying dimensions from these items, using principal components analyses (PCAs). This approach has been used elsewhere (e.g., Flouri and Ioakeimidi, 2018). We will now describe our five outcomes in turn.

*Self-esteem* was measured with five items of the Rosenberg self-esteem scale: “On the whole I am satisfied with myself,” “I feel that I have a number of good qualities,” “I am able to do things as well as most other people,” “I am a person of value,” and “I feel good about myself.” Items were coded from 1 (“strongly disagree”) to 4 (“strongly agree”) and the scale score was the mean of the five items. The Cronbach's alpha of the scale was 0.75.

*Happiness* was measured with six items: “How do you feel about (a) your schoolwork, (b) the way you look, (c) your family, (d) your friends, (e) the school you go to, and (f) your life as a whole?” Items were coded from 1 (“not at all happy”) to 7 (“completely happy”) and the scale score was the mean of the items. The Cronbach's alpha of the scale was 0.84.

*Positive and negative mood* were measured with six items on the experience of positive and negative feelings: “In the last 4 weeks, how often did you (a) feel happy, (b) feel worried, (c) feel sad, (d) feel scared, (e) laugh, (f) get angry?” Items were coded from 1 (“never”) to 5 (“almost always”). Because the positive and negative items do not necessarily belong to the same scale, we ran a PCA on the six items (after checking that the six items were suitable for PCA; KMO = 0.76). The PCA resulted in two principal components with eigenvalues > 1. We rotated the component solution, using Oblimin oblique rotation. The components were “positive mood” (with items “happy” and “laugh”; scores ranging from −5.82 to 1.63) and “negative mood” (with items “worried,” “sad,” “scared,” and “angry”; scores ranging from −2.48 to 6.36).

*Antisocial behaviour* was measured with seven items: “Have you ever been noisy or rude in a public place so that people complained or got you into trouble?”; “Have you ever taken something from a shop without paying for it?”; “Have you ever written things or sprayed paint on a building, fence or train or anywhere else where you shouldn't have?”; “Have you ever on purpose damaged anything in a public place that didn't belong to you, for example by burning, smashing or breaking things like cars, bus shelters and rubbish bins?”; “How often do you misbehave or cause trouble in class?” (1 “never” to 4 “all of the time”); “Have you ever missed school without your parents' permission even if only for half a day or a single lesson?”; and “How often do you hurt or pick on other children on purpose?” (1 “never” to 6 “most days”). We selected these items because they are covering different aspects of antisocial and delinquent behaviour. Similar items are used, for example, in the subscale “conduct problems” of both the Strengths and Difficulties Questionnaire (Goodman et al., 1998) and the Child Behavior Checklist (Achenbach and Rescorla, 2001). After checking that the seven items were suitable for PCA (KMO = 0.75), we performed a PCA on the seven items, using Oblimin oblique rotation. The PCA resulted in two components with eigenvalues > 1: “delinquent behaviour” (with items “shoplifting,” “spraying graffiti,” “damaging things,” and “truancy”; scores ranging from −0.64 to 11.11) and “antisocial behaviour” (with items “noisy/rude in public,” “misbehaving in class,” and “bullying other children”; scores ranging from −1.95 to 7.41). After visual inspection of the distributions of the components, we found that there was only little variance on the “delinquent behaviour” scale. Therefore, we decided to exclude it from further analyses, leaving us with five outcomes considered in this study.

We assessed the correlations between the five outcomes (all *p* < 0.001). Self-esteem was positively correlated with happiness (*r* = 0.45) and positive mood (*r* = 0.26), and negatively correlated with negative mood (*r* = −0.32) and antisocial behaviour (*r* = −0.16). Similarly, happiness was positively correlated with positive mood (*r* = 0.24), and negatively correlated with negative mood (*r* = −0.32) and antisocial behaviour (*r* = −0.23). Positive mood was negatively correlated with negative mood (*r* = −0.21) and antisocial behaviour (*r* = −0.12). Finally, negative mood was positively correlated with antisocial behaviour (*r* = 0.28).

#### Neighbourhood

*Neighbourhood greenspace* was measured with data from the Multiple Environmental Deprivation Index (MEDIx; https://cresh.org.uk/cresh-themes/environmental-deprivation/medix-and-medclass/). The MEDIx greenspace measure used data from the Coordination of Information on the Environment (CORINE; EEA., 2000) and the 2001 Generalised Land Use Database (GLUD; Office of the Deputy Prime Minister, 2001). CORINE is a land cover dataset from 2000 for the UK that was derived from remotely sensed satellite imagery. It does not capture greenspaces smaller than 1 ha. GLUD classifies land use across England at high geographic resolution into nine categories: greenspace, domestic gardens, fresh water, domestic buildings, non-domestic buildings, roads, paths, railways, and other. Richardson and Mitchell (2010) combined data from CORINE and GLUD to create a measure of the percentage of greenspace in every UK ward. The measure included all vegetated areas larger than 5 m^2^ (excluding domestic gardens), regardless of their accessibility (i.e., private or public). In the MCS, greenspace data were converted into deciles ranging from 1 “least green” to 10 “most green.”

*Neighbourhood air pollution* was measured with data on particulate matter concentrations < 10 micrometres (PM_10_) from the MEDIx. PM_10_ concentrations were measured as annual mean concentrations (μg/m^3^) for each UK ward. PM_10_ data were taken from 1-km grids (modelled from National Atmospheric Emissions Inventory data). Average PM_10_ concentrations covered the years 1999 to 2003 and were population weighted (using output area units). In the MCS, air pollution data were converted into deciles ranging from 1 “least polluted” to 10 “most polluted.”

*Neighbourhood deprivation* was measured with the MCS strata at Sweep 1: England-Advantaged, England-Disadvantaged, England-Ethnic Minority, Wales-Advantaged, Wales-Disadvantaged, Scotland-Advantaged, Scotland-Disadvantaged, Northern Ireland-Advantaged, and Northern Ireland-Disadvantaged. There were only two strata for Wales, Scotland, and Northern Ireland because the proportion of the population that belongs to ethnic minority groups was expected to be very small for these countries. For more information on stratification, please see Plewis (2007).

*Perceived area safety* was measured with a single item answered by the adolescents: “How safe is it to walk, play or hang out in this area during the day?”; 1 (“not at all safe”) to 4 (“very safe”).

*Availability of parks or playgrounds* was measured with a single item also answered by the adolescents: “Are there any parks or playgrounds in this area where children your age can play outdoors?” (no/yes).

#### Covariates

Family-level covariates were *maternal education* (University education; no/yes), *maternal depression* (measured at Sweep 1 with nine items of the Malaise inventory; scores ranging from 0 to 9, with higher scores indicating higher levels of depression), *intact family structure* (whether or not the child lived with their biological parent(s) continuously throughout Sweeps 1 to 5; no/yes), *home ownership* (whether the family owned its home; no/yes), and *access to a private garden* (no/yes). Child-level covariates were *sex* (male/female), *ethnicity* (White, Mixed, Indian, Pakistani and Bangladeshi, Black or Black British, or Other), *pubertal status* (started puberty; no/yes), and *physical activity*. The latter was measured with a single item answered by the adolescents: “How often do you play sports or active games inside or outside, not at school?”; 1 (“never”) to 5 (“most days”).

### Statistical Analysis

All analyses were run in Stata 15. We built three linear regression models for each of the five outcomes: a minimally adjusted model, a fully adjusted model, and a fully adjusted model with three interaction terms. The minimally adjusted model included neighbourhood greenspace, neighbourhood deprivation, sex, and age. The fully adjusted model added neighbourhood air pollution, availability of parks, perceived area safety, and family- and child-level covariates. The third model added the three interaction terms: garden access ^*^ greenspace, physical activity ^*^ greenspace, and area safety ^*^ greenspace. All models accounted for stratification and clustering and were weighted to account for selective attrition (using a study weight provided by the MCS). Under the assumption that missing data were missing at random (MAR), missing data on covariates and outcomes were imputed using multiple imputation by chained equations (MICE; Raghunathan et al., 2001). We generated 25 imputed datasets and used Rubin's combination rules to pool the obtained individual estimates into a single set of multiply imputed estimates (Rubin, 1987). Note that the majority of adolescents in our sample had complete data (82%). The highest missingness observed was for pubertal status (7%).

## Results

### Bias Analysis

We tested whether the analytic sample (*n* = 4,534) was different from the non-analytic sample (*n* = 14,709) on the study variables (see Table 1). On average, children in the analytic sample lived in less green and more polluted areas and were less likely to have access to a garden. However, children in the analytic sample were more likely to report that there were parks or playgrounds in the area where children of their age could play. Families in the analytic sample were more likely to own their home and to have been intact throughout.

**Table 1:** Bias analysis of study variables between analytic and non-analytic samples.

	**Analytic sample (** ***n*** **= 4534)**		**Non-analytic sample (** ***n*** **= 14,709)**		**Test**
**Continuous variables**					
	***N***	**M (SD)**	***N***	**M (SD)**	**F**
Self-esteem (1–4) (S5)	4,486	3.41 (0.44)	8,361	3.37 (0.44)	19.04^**^
Happiness (1–7) (S5)	4,515	5.97 (1.06)	8,405	5.92 (1.08)	7.81^**^
Antisocial behaviour (−1.95 to 7.41) (S5)	4,354	−0.05 (1.26)	8,053	0.03 (1.32)	10.32^**^
Positive mood (−5.82 to 1.63) (S5)	4,361	0.04 (1.1)	8,069	−0.02 (1.12)	7.36^**^
Negative mood (−2.48 to 6.36) (S5)	4,361	−0.02 (1.55)	8,069	0.01 (1.52)	0.78
Greenspace (1–10) (S5)	4,534	3.53 (2.1)	8,746	5.19 (2.84)	1,199.4^**^
Air pollution (1–10) (S5)	4,534	7.01 (2.74)	8,746	5.8 (3.09)	495.5^**^
Perceived area safety (1–4) (S5)	4,458	3.16 (0.64)	8,295	3.2 (0.65)	11.59^**^
Maternal depression (0–9) (S1)	4,321	1.63 (1.72)	13,482	1.72 (1.81)	9.21^**^
Physical activity (1–5) (S5)	4,498	4.37 (0.95)	8,381	4.39 (0.94)	1.23
Age (S5)	4,534	11.16 (0.33)	8,753	11.17 (0.33)	9.64^**^
**Categorical variables**					
	***N***	**%**	***N***	**%**	**Chi** ^**2**^
England-advantaged (S1)	1,210	26.7	3,618	24.6	8.06^**^
England-disadvantaged (S1)	1,164	25.7	3,641	24.8	1.56
England-ethnic minority (S1)	716	15.8	1,875	12.8	27.57^**^
Wales-advantaged (S1)	189	4.2	643	4.4	0.35
Wales-disadvantaged (S1)	490	10.8	1,438	9.8	4.09^*^
Scotland-advantaged (S1)	207	4.6	938	6.4	20.32^**^
Scotland-disadvantaged (S1)	213	4.7	978	6.7	22.72^**^
Northern Ireland-advantaged (S1)	127	2.8	596	4.1	15^**^
Northern Ireland-disadvantaged (S1)	218	4.8	982	6.7	20.68^**^
Available parks in the area (S5)	3,950	87.7	7,059	84.2	28.59^**^
Access to a garden (S5)	4,188	92.4	8,312	95.1	41.76^**^
Family owns its home (S5)	3,346	75	5,088	59.2	321.69^**^
Intact family structure (S1–S5)	3,519	77.6	10,456	71.1	73.65^**^
University education (mother) (S1–S5)	1,473	32.5	4,056	27.7	39.5^**^
Ethnicity White (S1)	3,559	78.5	12,184	83	43.83^**^
Ethnicity Mixed (S1)	133	2.9	461	3.1	0.47
Ethnicity Indian (S1)	176	3.9	321	2.2	39.78^**^
Ethnicity Pakistani and Bangladeshi (S1)	421	9.3	929	6.3	46.85^**^
Ethnicity Black or Black British (S1)	167	3.7	563	3.8	0.2
Ethnicity Other (S1)	78	1.7	226	1.5	0.75
Female (S1)	2254	49.7	7,095	48.2	3.03
Started puberty (S5)	2,625	62	5,147	62.5	0.32
Male (S5)	831	38.9	1,675	40.3	1.18
Female (S5)	1,794	85.5	3,472	85.1	0.17

*(S1), data from Sweep 1; (S5), data from Sweep 5; (S1–S5), data from across sweeps*.

*Ns, means, and %s are all unweighted*.

* *p < 0.05*,

** *p < 0.01*.

### Descriptive Statistics

In Table 1, we summarise descriptive statistics of our sample. On average, adolescents had high scores on self-esteem, happiness, and positive mood, and low scores on negative mood and antisocial behaviour. The great majority had access to a private garden. On average, adolescents perceived their neighbourhoods to be safe and reported high levels of physical activity. Compared to the distribution of wards in the UK, on average, our sample lived in less green and more polluted areas.

### Model Results

In Table 2, we summarise the minimally adjusted models. We did not find an association of greenspace with any of the five outcomes. Sex was the best predictor of emotional and behavioural adjustment: on average, girls had lower self-esteem, showed less antisocial behaviour, and reported higher levels of both positive and negative mood than boys. In Table 3, we summarise the fully adjusted models. Again, greenspace did not predict emotional or behavioural adjustment. Perceived area safety was a predictor of all outcomes. Sex remained a predictor of self-esteem, antisocial behaviour, and positive mood. Physical activity predicted most outcomes.

**Table 2:** Minimally adjusted regression models predicting self-esteem, happiness, positive mood, negative mood, and antisocial behaviour (*n* = 4,534).

	**Self-esteem**		**Happiness**		**Positive mood**		**Negative mood**		**Antisocial behaviour**	
	***b*** **(SE)**	**95% CI**	***b*** **(SE)**	**95% CI**	***b*** **(SE)**	**95% CI**	***b*** **(SE)**	**95% CI**	***b*** **(SE)**	**95% CI**
Greenspace	−0.002 (0.005)	(−0.011, 0.007)	−0.008 (0.01)	(−0.029, 0.013)	0.001 (0.012)	(−0.023, 0.024)	−0.01 (0.017)	(−0.044, 0.025)	−0.002 (0.012)	(−0.027, 0.022)
**Stratum (ref. England-advantaged)**										
England-disadvantaged	0.032 (0.022)	(−0.011, 0.075)	0.05 (0.052)	(−0.053, 0.153)	−0.066 (0.061)	(−0.186, 0.055)	0.008 (0.088)	(−0.164, 0.181)	0.134 (0.058)^*^	(0.019, 0.248)
England-ethnic minority	0.087 (0.031)^**^	(0.026, 0.147)	0.179 (0.062)^**^	(0.056, 0.301)	−0.002 (0.056)	(−0.112, 0.109)	−0.165 (0.121)	(−0.403, 0.072)	0.036 (0.094)	(−0.15, 0.222)
Wales-advantaged	0.031 (0.037)	(−0.041, 0.103)	0.103 (0.07)	(−0.035, 0.242)	0.01 (0.078)	(−0.144, 0.163)	−0.149 (0.106)	(−0.357, 0.06)	−0.062 (0.099)	(−0.257, 0.132)
Wales-disadvantaged	−0.017 (0.026)	(−0.067, 0.034)	0.06 (0.063)	(−0.064, 0.184)	0.042 (0.072)	(−0.099, 0.183)	−0.21 (0.089)^*^	(−0.386, −0.035)	0.077 (0.076)	(−0.073, 0.227)
Scotland-advantaged	0.039 (0.054)	(−0.067, 0.145)	0.11 (0.107)	(−0.101, 0.321)	0.15 (0.101)	(−0.049, 0.349)	−0.084 (0.117)	(−0.313, 0.145)	0.083 (0.103)	(−0.12, 0.285)
Scotland-disadvantaged	0.096 (0.042)^*^	(0.012, 0.179)	0.04 (0.1)	(−0.148, 0.229)	0.091 (0.097)	(−0.1, 0.283)	−0.165 (0.116)	(−0.393, 0.062)	−0.118 (0.120)	(−0.354, 0.118)
Northern Ireland-advantaged	0.066 (0.035)	(−0.004, 0.136)	0.35 (0.068)^**^	(0.216, 0.483)	0.204 (0.117)	(−0.027, 0.435)	−0.241 (0.147)	(−0.53, 0.048)	−0.088 (0.128)	(−0.341, 0.164)
Northern Ireland-disadvantaged	0.072 (0.037)	(−0.001, 0.144)	0.281 (0.083)^**^	(0.119, 0.444)	0.179 (0.101)	(−0.02, 0.379)	−0.388 (0.102)^**^	(−0.589, −0.187)	0.064 (0.111)	(−0.155, 0.284)
Age	0.026 (0.028)	(−0.028, 0.081)	0.056 (0.06)	(−0.063, 0.174)	0.131 (0.065)^*^	(0.003, 0.258)	−0.075 (0.084)	(−0.24, 0.091)	0.06 (0.077)	(−0.091, 0.211)
Female	−0.064 (0.017)^**^	(−0.098, −0.031)	0.011 (0.037)	(−0.062, 0.084)	0.282 (0.042)^**^	(0.199, 0.365)	0.123 (0.057)^*^	(0.012, 0.234)	−0.558 (0.044)^**^	(−0.645, −0.472)
Constant	3.113 (0.306)^**^	(2.511, 3.715)	5.285 (0.666)^**^	(3.974, 6.597)	−1.586 (0.717)^*^	(−2.997, −0.175)	0.891 (0.939)	(−0.958, 2.74)	−0.443 (0.868)	(−2.152, 1.266)

*Estimates are pooled estimates of 25 imputed datasets*.

* *p < 0.05*,

** *p < 0.01*.

**Table 3:** Fully adjusted regression models predicting self-esteem, happiness, positive mood, negative mood, and antisocial behaviour (*n* = 4,534).

	**Self-esteem**		**Happiness**		**Positive mood**		**Negative mood**		**Antisocial behaviour**	
	***b*** **(SE)**	**95% CI**	***b*** **(SE)**	**95% CI**	***b*** **(SE)**	**95% CI**	***b*** **(SE)**	**95% CI**	***b*** **(SE)**	**95% CI**
Greenspace	−0.001 (0.005)	(−0.01, 0.009)	−0.005 (0.011)	(−0.026, 0.016)	−0.002 (0.013)	(−0.027, 0.023)	−0.002 (0.018)	(−0.036, 0.033)	−0.004 (0.014)	(−0.031, 0.024)
**Stratum (ref. England-advantaged)**										
England-disadvantaged	0.059 (0.021)^**^	(0.018, 0.101)	0.097 (0.05)	(−0.002, 0.197)	−0.019 (0.062)	(−0.14, 0.103)	−0.068 (0.087)	(−0.238, 0.102)	0.027 (0.057)	(−0.085, 0.14)
England-ethnic minority	0.069 (0.034)^*^	(0.002, 0.137)	0.052 (0.072)	(−0.09, 0.194)	0.096 (0.088)	(−0.078, 0.27)	−0.265 (0.137)	(−0.536, 0.005)	−0.092 (0.106)	(−0.3, 0.116)
Wales-advantaged	0.052 (0.037)	(−0.02, 0.125)	0.151 (0.072)^*^	(0.008, 0.294)	0.039 (0.075)	(−0.108, 0.186)	−0.201 (0.109)	(−0.417, 0.014)	−0.122 (0.105)	(−0.328, 0.083)
Wales-disadvantaged	0.033 (0.026)	(−0.019, 0.085)	0.17 (0.066)^**^	(0.04, 0.3)	0.095 (0.076)	(−0.054, 0.245)	−0.318 (0.097)^**^	(−0.51, −0.126)	−0.08 (0.08)	(−0.238, 0.078)
Scotland-advantaged	0.043 (0.052)	(−0.06, 0.146)	0.117 (0.105)	(−0.089, 0.324)	0.147 (0.111)	(−0.071, 0.366)	−0.063 (0.146)	(−0.35, 0.224)	−0.011 (0.125)	(−0.257, 0.235)
Scotland-disadvantaged	0.139 (0.043)^**^	(0.054, 0.224)	0.13 (0.102)	(−0.071, 0.33)	0.144 (0.106)	(−0.065, 0.352)	−0.257 (0.142)	(−0.536, 0.022)	−0.291 (0.136)^*^	(−0.559, −0.022)
Northern Ireland-advantaged	0.049 (0.038)	(−0.026, 0.123)	0.299 (0.081)^**^	(0.14, 0.458)	0.177 (0.116)	(−0.051, 0.405)	−0.17 (0.154)	(−0.474, 0.134)	−0.077 (0.122)	(−0.317, 0.163)
Northern Ireland-disadvantaged	0.089 (0.037)^*^	(0.016, 0.161)	0.321 (0.084)^**^	(0.155, 0.486)	0.164 (0.102)	(−0.037, 0.364)	−0.386 (0.115)^**^	(−0.612, −0.161)	−0.065 (0.113)	(−0.287, 0.156)
Air pollution	0.008 (0.005)	(−0.001, 0.017)	0.013 (0.01)	(−0.007, 0.033)	0.012 (0.012)	(−0.011, 0.036)	−0.013 (0.017)	(−0.046, 0.019)	−0.024 (0.014)	(−0.052, 0.004)
Availability of parks	0.026 (0.027)	(−0.027, 0.08)	−0.031 (0.052)	(−0.133, 0.071)	0.058 (0.066)	(−0.072, 0.188)	0.039 (0.116)	(−0.188, 0.267)	0.012 (0.074)	(−0.133, 0.157)
Perceived area safety	0.101 (0.012)^**^	(0.076, 0.126)	0.242 (0.03)^**^	(0.183, 0.3)	0.17 (0.033)^**^	(0.105, 0.235)	−0.402 (0.047)^**^	(−0.495, −0.309)	−0.167 (0.038)^**^	(−0.242, −0.092)
Access to garden	−0.031 (0.031)	(−0.092, 0.029)	−0.049 (0.072)	(−0.191, 0.092)	0.116 (0.106)	(−0.093, 0.326)	−0.108 (0.112)	(−0.329, 0.112)	−0.053 (0.108)	(−0.267, 0.16)
Family owns its home	0.031 (0.022)	(−0.013, 0.075)	0.076 (0.05)	(−0.022, 0.173)	0.004 (0.06)	(−0.114, 0.122)	−0.024 (0.085)	(−0.19, 0.143)	−0.177 (0.069)^*^	(−0.313, −0.04)
Mother has University education	0.003 (0.017)	(−0.031, 0.037)	−0.031 (0.04)	(−0.109, 0.047)	−0.065 (0.042)	(−0.147, 0.018)	0.042 (0.058)	(−0.073, 0.157)	0.008 (0.054)	(−0.099, 0.115)
Maternal depression	−0.006 (0.005)	(−0.015, 0.003)	−0.02 (0.011)	(−0.041, 0.001)	−0.009 (0.012)	(−0.032, 0.014)	0.027 (0.016)	(−0.005, 0.059)	0.032 (0.014)^*^	(0.005, 0.059)
Intact family structure	0.066 (0.021)^**^	(0.025, 0.106)	0.157 (0.052)^**^	(0.055, 0.259)	0.063 (0.06)	(−0.056, 0.182)	−0.113 (0.076)	(−0.262, 0.037)	−0.175 (0.065)^**^	(−0.303, −0.046)
Started puberty	0.001 (0.017)	(−0.031, 0.034)	−0.057 (0.045)	(−0.146, 0.032)	0.018 (0.051)	(−0.082, 0.117)	0.016 (0.068)	(−0.118, 0.149)	0.042 (0.053)	(−0.062, 0.146)
Physical activity	0.072 (0.01)^**^	(0.053, 0.091)	0.102 (0.018)^**^	(0.066, 0.138)	0.144 (0.025)^**^	(0.096, 0.193)	−0.059 (0.033)	(−0.125, 0.006)	0.051 (0.025)^*^	(0.002, 0.1)
**Ethnicity (ref. White)**										
Mixed	0.035 (0.063)	(−0.088, 0.158)	0.104 (0.109)	(−0.111, 0.318)	0.042 (0.155)	(−0.262, 0.346)	−0.111 (0.179)	(−0.464, 0.242)	0.027 (0.118)	(−0.205, 0.259)
Indian	−0.02 (0.046)	(−0.111, 0.072)	0.246 (0.087)^**^	(0.075, 0.417)	−0.086 (0.101)	(−0.285, 0.112)	−0.156 (0.155)	(−0.462, 0.149)	−0.108 (0.117)	(−0.34, 0.123)
Pakistani and Bangladeshi	0.033 (0.031)	(−0.027, 0.094)	0.203 (0.077)^**^	(0.052, 0.354)	−0.129 (0.092)	(−0.31, 0.053)	0.127 (0.134)	(−0.137, 0.391)	0.184 (0.111)	(−0.034, 0.403)
Black or Black British	0.021 (0.041)	(−0.06, 0.103)	0.153 (0.101)	(−0.045, 0.352)	−0.091 (0.143)	(−0.372, 0.191)	0.095 (0.17)	(−0.241, 0.43)	0.019 (0.101)	(−0.18, 0.218)
Other	0.198 (0.051)^**^	(0.098, 0.297)	0.273 (0.114)^*^	(0.049, 0.498)	−0.113 (0.152)	(−0.413, 0.187)	0.106 (0.194)	(−0.277, 0.488)	−0.095 (0.117)	(−0.325, 0.135)
Age	0.016 (0.028)	(−0.038, 0.071)	0.054 (0.06)	(−0.065, 0.172)	0.105 (0.065)	(−0.024, 0.234)	−0.067 (0.083)	(−0.231, 0.097)	0.042 (0.074)	(−0.104, 0.189)
Female	−0.049 (0.017)^**^	(−0.082, −0.016)	0.064 (0.042)	(−0.018, 0.146)	0.308 (0.049)^**^	(0.211, 0.404)	0.092 (0.069)	(−0.043, 0.228)	−0.573 (0.051)^**^	(−0.672, −0.473)
Constant	2.445 (0.317)^**^	(1.821, 3.069)	3.9 (0.698)^**^	(2.527, 5.273)	−2.753 (0.716)^**^	(−4.162, −1.343)	2.551 (0.973)^**^	(0.635, 4.468)	0.514 (0.859)	(−1.177, 2.204)

*Estimates are pooled estimates of 25 imputed datasets*.

* *p < 0.05*,

** *p < 0.01*.

**Figure 1:**
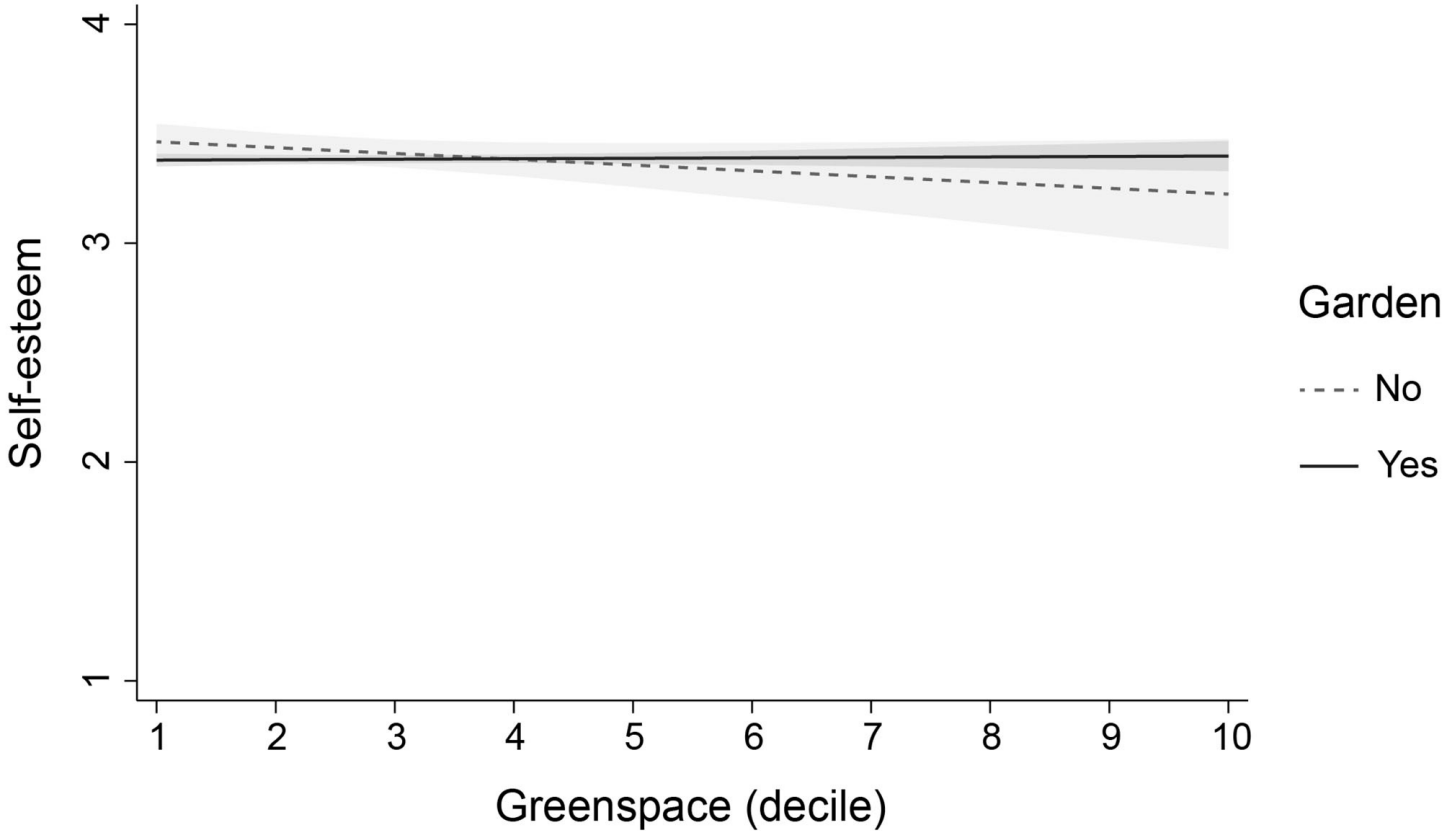
Modification of the effect of greenspace on self-esteem by garden access. This plot illustrates the modifying role of garden access in the association of greenspace with self-esteem (linear predictions). Plots are based on non-imputed data. Shaded areas are 95% confidence intervals.

**Figure 2:**
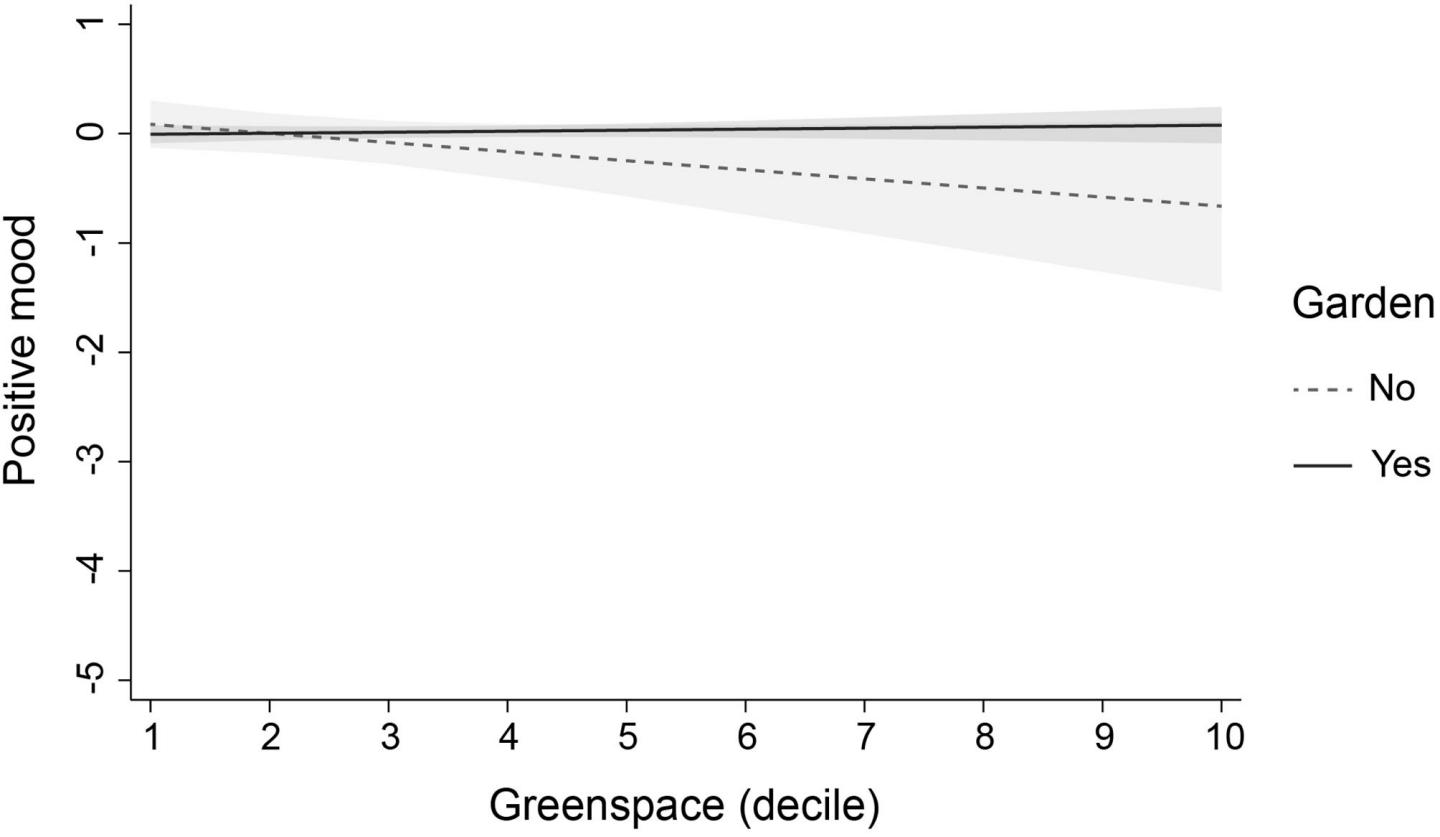
Modification of the effect of greenspace on positive mood by garden access. This plot illustrates the modifying role of garden access in the association of greenspace with positive mood (linear predictions). Plots are based on non-imputed data. Shaded areas are 95% confidence intervals.

**Figure 3:**
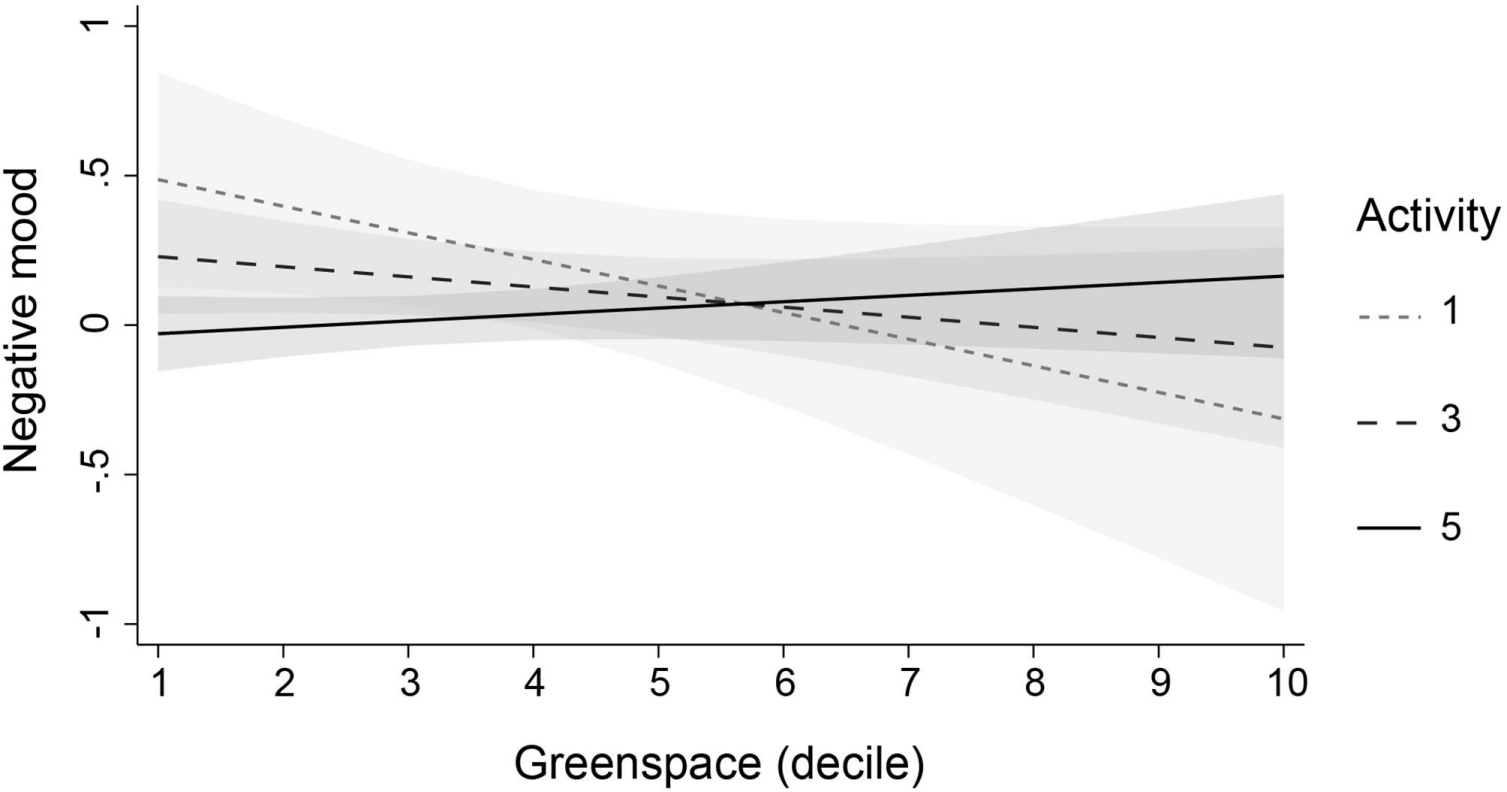
Moderation of the effect of greenspace on negative mood by physical activity. This plot illustrates the moderating role of physical activity in the association of greenspace with negative mood (linear predictions). Plots are based on non-imputed data. Shaded areas are 95% confidence intervals. Physical activity levels ranged from 1 “never” to 5 “most days.” Please note that, for visual clarity, first, the y-axis does not cover the whole scale of negative mood, and second, lines for activity levels 2 and 4 were omitted.

**Figure 4:**
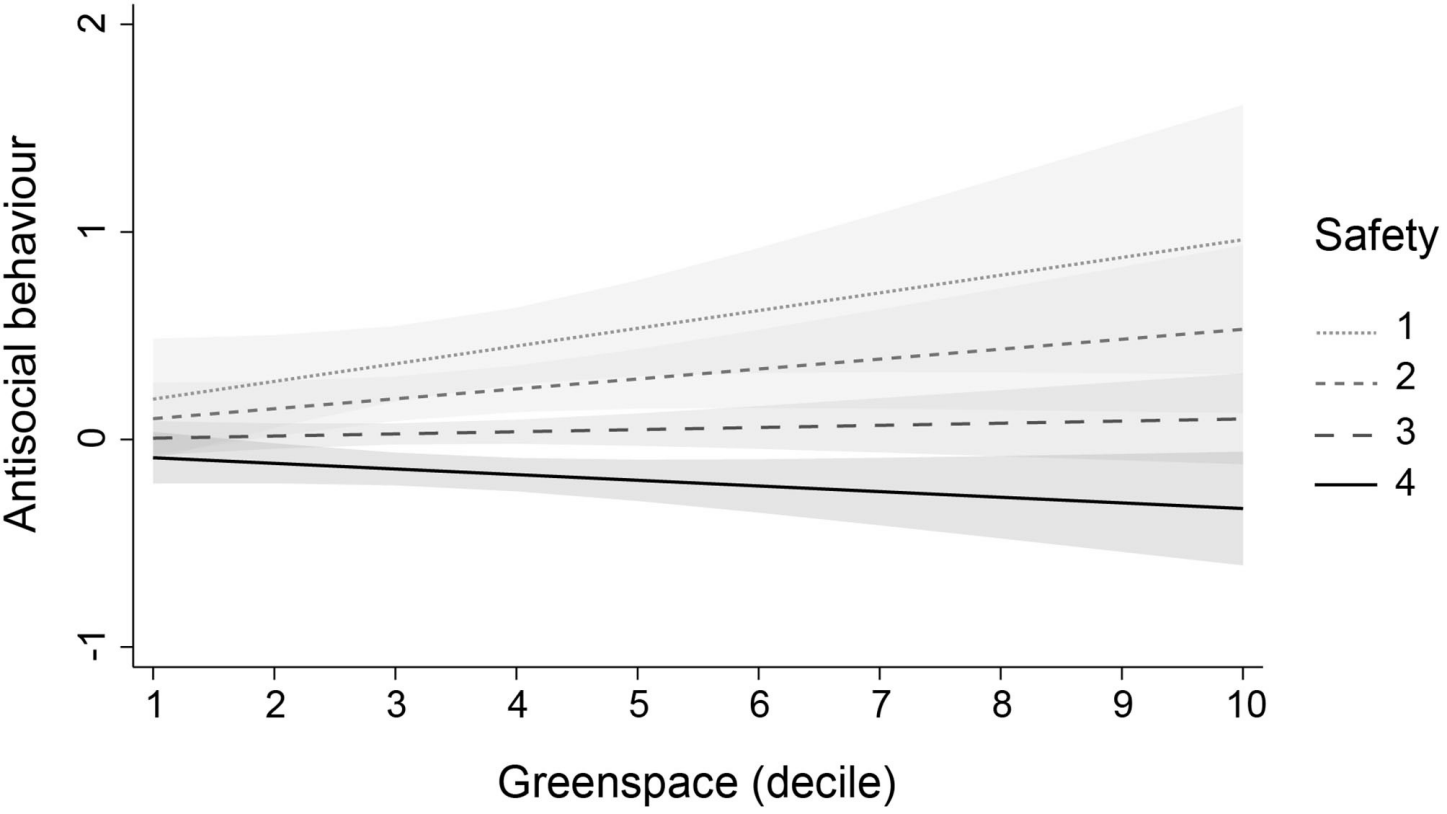
Moderation of the effect of greenspace on antisocial behaviour by area safety. This plot illustrates the moderating role of perceived area safety in the association of greenspace with antisocial behaviour (linear predictions). Plots are based on non-imputed data. Shaded areas are 95% confidence intervals. Perceived area safety ranged from 1 “not at all safe” to 4 “very safe.” Please note that, for visual clarity, the y-axis does not cover the whole scale of antisocial behaviour.

To investigate whether the effect of greenspace was modified or moderated by other variables, we added three interaction terms to each of the fully adjusted models. We found four significant interactions (not reported in tables). First, access to a private garden modified the effect of greenspace on adolescents' self-esteem (*b* = 0.035, SE = 0.016, *p* = 0.028, 95% CI: 0.004, 0.066) and positive mood (*b* = 0.149, SE = 0.067, *p* = 0.027, 95% CI: 0.017, 0.28): in adolescents without a garden, higher levels of neighbourhood greenspace were associated with lower levels of self-esteem and positive mood. Second, physical activity moderated the effect of greenspace on negative mood (*b* = 0.033, SE = 0.014, *p* = 0.019, 95% CI: 0.005, 0.061): in adolescents who reported lower levels of physical activity, higher levels of greenspace were associated with lower levels of negative mood. Finally, perceived area safety moderated the effect of greenspace on antisocial behaviour (*b* = −0.039, SE = 0.019, *p* = 0.035, 95% CI: −0.076, −0.003): in adolescents who perceived their areas to be unsafe, higher levels of greenspace were associated with higher levels of antisocial behaviour. The interactions are illustrated in Figures 1–4. Note that the figures are based on non-imputed data and may deviate slightly from results reported in-text.

## Discussion

We investigated the role of neighbourhood greenspace in emotional and behavioural outcomes in young adolescents from urban areas in the UK, using data from the MCS. We did not find a main effect of greenspace on any of the five outcomes considered, but we did find interaction effects. We will now discuss our findings in the context of the previous literature and our study's limitations.

### Main Findings

We did not find a main effect of greenspace on any of the five outcomes. This was unexpected, especially in light of several previous studies that found associations of greenspace with a number of related outcomes in adolescents, including mood (Li et al., 2018), stress (Feda et al., 2015), and aggression (Younan et al., 2016). Yet, this is not the first study to report null results (e.g., Mueller et al., 2019; Weeland et al., 2019a), and our findings do fit in the mixed body of evidence. However, considering that Flouri et al. (2019) did find an effect of greenspace on spatial working memory, using data on the same cohort at the same age, it would be wrong to conclude that greenspace does not play a role in the mental health and well-being of young adolescents. The question is why we did not find an association in our study. There are two possible explanations for this: first, our measures of exposure and outcomes had limitations that may explain our null findings at least partly. We will describe these limitations in detail below. Second, it is possible that the effect of greenspace on mental health and well-being is conditional. We tested this and, indeed, found evidence for modification/moderation by garden access, physical activity, and perceived area safety.

First, we found that higher levels of greenspace in the neighbourhood were associated with lower levels of self-esteem and positive mood, but only in adolescents without a garden. This finding was contrary to our expectation that public greenspace would have a protective function for those without access to private greenspace. A possible explanation for our finding may be that families in the UK without access to a garden are more likely to be disadvantaged. Therefore, adolescents without a garden (likely from disadvantaged families) who live in green neighbourhoods (likely affluent) may feel relatively deprived. This is what the theory of relative deprivation would predict (Stouffer et al., 1949). According to this theory, being relatively deprived in comparison to a reference group causes stress (Winkleby et al., 2006), which can affect health negatively (Åberg Yngwe et al., 2003). A meta-analysis of the impact of relative deprivation on a range of outcomes provided evidence that one's perception of their relative injustice compared to a well-defined reference group (e.g., school, neighbourhood, or other) can have a significant impact on mental health (Smith et al., 2012). Indeed, in the UK, adolescents' perceived family social status (or subjective socio-economic status) has been linked to self-esteem, life satisfaction, and mental health problems (Quon and McGrath, 2014; Bannink et al., 2016; Rivenbark et al., 2020). However, without MCS data on the adolescents' perceived position compared to their neighbours', this explanation remains speculative. We should also note that we did not find the same effect for happiness or negative mood, although these are outcomes also related to emotional well-being.

Second, we found that higher levels of greenspace in the neighbourhood were associated with lower levels of negative mood, but only in adolescents who reported little engagement in sports or active games outside of school. We hypothesised that more active adolescents may be exposed more and, therefore, may benefit more from nearby greenspaces than their counterparts. However, our finding suggests the opposite. While adolescents who reported higher levels of physical activity were not affected by neighbourhood greenspace, adolescents who reported lower levels of physical activity seemed to benefit from high levels of greenspace and seemed to be negatively affected by lower levels of greenspace. This suggests a protective function of greenspace for adolescents who do not usually play sports or active games in their free time. A possible explanation for why more active adolescents did not seem to benefit from greenspace in their neighbourhoods would be that the question about physical activity did not specify *where* adolescents were active except for “not at school.” Adolescents who report high levels of physical activity may not be active in their neighbourhoods, but, for example, may be active participating in formal sports outside their area. Less active adolescents, on the other hand, may spend more time in their neighbourhoods and may, therefore, benefit more from nearby greenspaces. Future studies should further explore the role of physical activity, ideally with a more specific (and objective) measure of physical activity.

Third, we found that higher levels of neighbourhood greenspace were linked to higher levels of antisocial behaviour, but only in adolescents who perceived their areas to be unsafe. We expected that adolescents who perceived their areas to be safe would benefit more from greenspaces than adolescents who perceived their areas to be unsafe. Yet, our finding suggests that adolescents who perceive their areas to be unsafe may not only be unaffected but, indeed, negatively affected by neighbourhood greenspace. A possible explanation for this would be that adolescents who live in unsafe and likely more deprived neighbourhoods may be exposed to more antisocial behaviour. Higher levels of (potentially low-quality) greenspace in these areas may increase the risk of adopting such behaviours by offering opportunities to loiter and to engage in antisocial acts unmonitored. Again, future research is needed to explore this further.

### Study Limitations

We need to view our results in the light of our study's limitations. First, there were limitations to our measure of greenspace. We used a measure of neighbourhood greenspace at ward level. A ward is a unit of administrative geography in the UK that can be large and varying in size. As we did not know where exactly an adolescent lived in a ward, our greenspace measure may not have captured their true exposure. An adolescent may live in a ward that falls into a low greenspace decile but very close to a ward that falls into a high greenspace decile. Similarly, an adolescent may live in a ward with generally low levels of greenspace but close to a large urban park. In both cases, actual exposure to greenspace would be more than suggested by our measure. Further, greenspace was measured with data from 2000 and 2001—about 10 years prior to when our outcomes were measured. Although levels of greenspace in the UK are not expected to change much over a decade, this may have led to measurement error. Therefore, we must consider exposure misclassification to have attenuated effects. We should also note that we used a measure of greenspace *quantity*. Other dimensions, however, such as quality, proximity, and use, may also be important for the outcomes considered in our study. Future studies would benefit from a multidimensional approach.

Our outcomes also had limitations. First, all outcomes were self-reported. This may be an advantage for emotional outcomes because the adolescents themselves may know better how they feel than their parents or teachers. However, in the case of antisocial behaviour, data could be biased, for example, toward social expectations. Second, only one of the outcomes, the Rosenberg self-esteem scale, is a well-established and validated measure. Reliability and validity are important concepts in psychology and indicate the quality of a measure (i.e., whether it measures something consistently and accurately). In this study, we were relying on the data available in the MCS. Therefore, some of our outcomes had to be derived from the items available. For the happiness scale, it was clear that the six items measured the same construct (i.e., happiness), and this scale had already been used in the literature. For positive mood, negative mood, and antisocial behaviour scales, we took steps to ensure that these outcomes were meaningful. First, by basing the item selection on the content of the items and on comparisons with related, established questionnaires, we made sure that each outcome measured a certain construct. Second, by running a PCA on the selected items, we made sure that the items did share an underlying component/construct. Third, we assessed the correlations among the outcomes which were all in the expected directions. Therefore, we expect that our measures were able to capture (at least aspects of the) intended constructs. Nonetheless, it is important to keep in mind that some of our outcomes may be limited, for example, by a small number of items. Future studies would benefit from using established, validated measures of mental health and well-being.

We should note two additional limitations. First, we used observational and cross-sectional data. Therefore, inferences about causality must be made with caution. We minimised this problem by controlling for neighbourhood and family confounders, but we cannot rule out confounding by other unknown or unmeasured factors. Second, our analytic sample was selective because we restricted it to 11-year-old adolescents from urban areas who had never moved. This limits the generalisability of our results. In particular, we cannot make inferences about older adolescents, about adolescents from rural areas in the UK, or about adolescents who have moved home during their childhood.

## Conclusion

We investigated the role of neighbourhood greenspace in emotional and behavioural outcomes of young adolescents from urban UK. Greenspace did not have a main effect on any of the five outcomes considered. However, we did find interaction effects that are worth exploring further in future studies. Our study's limitations prevent us from drawing general conclusions or conclusions about causality. However, what we would like the reader to take away from our study is that we may, indeed, not be able to draw universal conclusions. Many other factors play a role, and, for a better understanding of the role of neighbourhood greenspace in adolescent mental health and well-being, we must pay attention to modifying and moderating factors. This could have implications for policymaking, interventions, and urban planning and design.

## Data Availability Statement

Publicly available data were used in this study. These data can be downloaded from the UK Data Service: https://ukdataservice.ac.uk/.

## Ethics Statement

Ethical approval has been obtained by those responsible for the MCS. The MCS has also sought written informed consent from parents for their participation and the participation of their children. Details can be found on the MCS website: https://cls.ucl.ac.uk/cls-studies/millennium-cohort-study/.

## Author Contributions

MM and EF contributed to conception, design of the study, and edited the manuscript for submission. MM performed the statistical analysis and wrote the first draft of the manuscript. Both authors contributed to the article and approved the submitted version.

## Funding

This research was funded by a grant from the Economic and Social Research Council to EF (ES/N007921/1) and by a PhD studentship award to MM from the Leverhulme Doctoral Training Programme for the Ecological Study of the Brain (DS-2017-026).

## Conflict of Interest

The authors declare that the research was conducted in the absence of any commercial or financial relationships that could be construed as a potential conflict of interest.

## Publisher's Note

All claims expressed in this article are solely those of the authors and do not necessarily represent those of their affiliated organizations, or those of the publisher, the editors and the reviewers. Any product that may be evaluated in this article, or claim that may be made by its manufacturer, is not guaranteed or endorsed by the publisher.

## References

[B1] Åberg Yngwe M. Fritzell J. Lundberg O. Diderichsen F. Burström B. (2003). Exploring relative deprivation: is social comparison a mechanism in the relation between income and health? Soc. Sci. Med. 57, 1463–1473. 10.1016/S0277-9536(02)00541-5 12927476

[B2] Achenbach T. M. Rescorla L. A. (2001). Manual for the ASEBA School-Age Forms and Profiles. Child Behavior Checklist for Ages 6–18. An Integrated System of Multi-Informant Assessment. Burlington: ASBEA

[B3] Aminzadeh K. Denny S. Utter J. Milfont T. L. Ameratunga S. Teevale T.. (2013). Neighbourhood social capital and adolescent self-reported wellbeing in New Zealand: a multilevel analysis. Soc. Sci. Med. 84, 13–21. 10.1016/j.socscimed.2013.02.012 23517699

[B4] Astell-Burt T. Feng X. Kolt G. S. (2013). Mental health benefits of neighbourhood green space are stronger among physically active adults in middle-to-older age: evidence from 260,061 Australians. Prev. Med. 57, 601–606. 10.1016/j.ypmed.2013.08.017 23994648

[B5] Balseviciene B. Sinkariova L. Grazuleviciene R. Andrusaityte S. Uzdanaviciute I. Dedele A.. (2014). Impact of residential greenness on preschool children's emotional and behavioral problems. Int. J. Environ. Res. Public Health 11, 6757–6770. 10.3390/ijerph110706757 24978880 PMC4113842

[B6] Bannink R. Pearce A. Hope S. (2016). Family income and young adolescents' perceived social position: associations with self-esteem and life satisfaction in the UK Millennium Cohort Study. Arch. Dis. Child. 101, 917–921. 10.1136/archdischild-2015-309651 26957529 PMC5050283

[B7] Bloemsma L. D. Gehring U. Klompmaker J. O. Hoek G. Janssen N. A. H. Smit H. A.. (2018). Green space visits among adolescents: frequency and predictors in the PIAMA birth cohort study. Environ. Health Perspect. 126, 1–9. 10.1289/EHP2429 29714963 PMC6071798

[B8] Bowler D. E. Buyung-Ali L. M. Knight T. M. Pullin A. S. (2010). A systematic review of evidence for the added benefits to health of exposure to natural environments. BMC Public Health 10:456. 10.1186/1471-2458-10-456 20684754 PMC2924288

[B9] Brennan P. A. Hammen C. Katz A. R. Le Brocque R. M. (2002). Maternal depression, paternal psychopathology, and adolescent diagnostic outcomes. J. Consult. Clin. Psychol. 70, 1075–1085. 10.1037/0022-006X.70.5.1075 12362958

[B10] Brown J. Cohen P. Johnson J. G. Smailes E. M. (1999). Childhood abuse and neglect: specificity of effects on adolescent and young adult depression and suicidality. J. Am. Acad. Child Adolesc. Psychiatry 38, 1490–1496. 10.1097/00004583-199912000-00009 10596248

[B11] Card N. A. Stucky B. D. Sawalani G. M. Little T. D. (2008). Direct and indirect aggression during childhood and adolescence: a meta-analytic review of gender differences, intercorrelations, and relations to maladjustment. Child Dev. 79, 1185–1229. 10.1111/j.1467-8624.2008.01184.x 18826521

[B12] Chong S. Lobb E. Khan R. Abu-Rayya H. Byun R. Jalaludin B. (2013). Neighbourhood safety and area deprivation modify the associations between parkland and psychological distress in Sydney, Australia. BMC Public Health 13:422. 10.1186/1471-2458-13-422 23635303 PMC3643863

[B13] de Bell S. White M. Griffiths A. Darlow A. Taylor T. Wheeler B.. (2020). Spending time in the garden is positively associated with health and wellbeing: results from a national survey in England. Landsc. Urban Plan. 200, 103836. 10.1016/j.landurbplan.2020.103836

[B14] Demuth S. Brown S. L. (2004). Family structure, family processes, and adolescent delinquency: the significance of parental absence versus parental gender. J. Res. Crime Delinq. 41, 58–81. 10.1177/0022427803256236

[B15] Department of Health (2013). Annual report of the Chief Medical Officer: Our Children Deserve Better. Annual Report of the Chief Medical Officer 2012. Available online at: https://www.gov.uk/government/publications/chief-medical-officers-annual-report-2012-our-children-deserve-better-prevention-pays (accessed August 29, 2021).

[B16] Eamon M. K. (2001). Poverty, parenting, peer, and neighborhood influences on young adolescent antisocial behavior. J. Soc. Serv. Res. 28, 1–23. 10.1300/J079v28n01_01

[B17] EEA. (2000). CORINE land cover 2000. Available online at: http://www.eea.europa.eu/publications/COR0-landcover (accessed August 29, 2021).

[B18] Feda D. M. Seelbinder A. Baek S. Raja S. Yin L. Roemmich J. N. (2015). Neighbourhood parks and reduction in stress among adolescents: results from Buffalo, New York. Indoor Built Environ. 24, 631–639. 10.1177/1420326X14535791

[B19] Fergusson D. M. Horwood L. J. Ridder E. M. Beautrais A. L. (2005). Subthreshold depression in adolescence and mental health outcomes in adulthood. Arch. Gen. Psychiatry 62, 66–72. 10.1001/archpsyc.62.1.66 15630074

[B20] Flouri E. Ioakeimidi S. (2018). Maternal depressive symptoms in childhood and risky behaviours in early adolescence. Eur. Child Adolesc. Psychiatry 27, 301–308. 10.1007/s00787-017-1043-6 28905111 PMC5852181

[B21] Flouri E. Midouhas E. Joshi H. (2014). The role of urban neighbourhood green space in children's emotional and behavioural resilience. J. Environ. Psychol. 40, 179–186. 10.1016/j.jenvp.2014.06.007

[B22] Flouri E. Papachristou E. Midouhas E. (2019). The role of neighbourhood greenspace in children's spatial working memory. Br. J. Educ. Psychol. 89, 359–373. 10.1111/bjep.12243 30187470 PMC6563484

[B23] Ford J. L. Rechel M. (2012). Parental perceptions of the neighborhood context and adolescent depression. Public Health Nurs. 29, 390–402. 10.1111/j.1525-1446.2012.01015.x 22924562

[B24] Ford T. Parker C. (2016). Emotional and behavioural difficulties and mental (ill)health. Emot. Behav. Difficult. 21, 1–7. 10.1080/13632752.2016.113930029234521

[B25] Gómez J. E. Johnson B. A. Selva M. Sallis J. F. (2004). Violent crime and outdoor physical activity among inner-city youth. Prev. Med. 39, 876–881. 10.1016/j.ypmed.2004.03.019 15475019

[B26] Goodman A. Joyce R. Smith J. P. (2011). The long shadow cast by childhood physical and mental problems on adult life. Proc. Natl. Acad. Sci. USA. 108, 6032–6037. 10.1073/pnas.1016970108 21444801 PMC3076863

[B27] Goodman R. Meltzer H. Bailey V. (1998). The strengths and difficulties questionnaire: a pilot study on the validity of the self-report version. Eur. Child Adolesc. Psychiatry 7, 125–130. 10.1007/s007870050057 9826298

[B28] Griffin K. W. Botvin G. J. Scheier L. M. Diaz T. Miller N. L. (2000). Parenting practices as predictors of substance use, delinquency, and aggression among urban minority youth: moderating effects of family structure and gender. Psychol. Addict. Behav. 14, 174–184. 10.1037/0893-164X.14.2.174 10860116 PMC3962786

[B29] Hartig T. Mitchell R. De Vries S. Frumkin H. (2014). Nature and health. Annu. Rev. Public Health 35, 207–228. 10.1146/annurev-publhealth-032013-182443 24387090

[B30] Heizomi H. Allahverdipour H. Asghari Jafarabadi M. Safaian A. (2015). Happiness and its relation to psychological well-being of adolescents. Asian J. Psychiatr. 16, 55–60. 10.1016/j.ajp.2015.05.037 26059325

[B31] Johnson J. G. Cohen P. Kasen S. (2009). Minor depression during adolescence and mental health outcomes during adulthood. Br. J. Psychiatry 195, 264–265. 10.1192/bjp.bp.108.054239 19721119 PMC2801821

[B32] Jones A. Hillsdon M. Coombes E. (2009). Greenspace access, use, and physical activity: understanding the effects of area deprivation. Prev. Med. 49, 500–505. 10.1016/j.ypmed.2009.10.012 19857513 PMC3748371

[B33] Kelly Y. Patalay P. Montgomery S. Sacker A. (2016). BMI development and early adolescent psychosocial well-being: UK Millennium Cohort Study. Pediatrics 138:967. 10.1542/peds.2016-0967 27940679 PMC5127062

[B34] Kelly Y. Zilanawala A. Booker C. Sacker A. (2018). Social media use and adolescent mental health: findings from the UK millennium cohort study. EClinicalMedicine 6, 59–68. 10.1016/j.eclinm.2018.12.005 31193561 PMC6537508

[B35] Kim-Cohen J. Caspi A. Moffitt T. E. Harrington H. L. Milne B. J. Poulton R. (2003). Prior juvenile diagnoses in adults with mental disorder: developmental follow-back of a prospective-longitudinal cohort. Arch. Gen. Psychiatry 60, 709–717. 10.1001/archpsyc.60.7.709 12860775

[B36] Li D. Deal B. Zhou X. Slavenas M. Sullivan W. C. (2018). Moving beyond the neighborhood: daily exposure to nature and adolescents' mood. Landsc. Urban Plan. 173, 33–43. 10.1016/j.landurbplan.2018.01.009

[B37] Li M. Johnson S. B. Musci R. J. Riley A. W. (2017). Perceived neighborhood quality, family processes, and trajectories of child and adolescent externalizing behaviors in the United States. Soc. Sci. Med. 192, 152–161. 10.1016/j.socscimed.2017.07.027 28835338

[B38] Lin B. B. Fuller R. A. Bush R. Gaston K. J. Shanahan D. F. (2014). Opportunity or orientation? Who uses urban parks and why. PLoS ONE 9:87422. 10.1371/journal.pone.0087422 24489913 PMC3906185

[B39] Maat K. de Vries P. (2006). The influence of the residential environment on green-space travel: testing the compensation hypothesis. Environ. Plan. A 38, 2111–2127. 10.1068/a37448

[B40] Markevych I. Schoierer J. Hartig T. Chudnovsky A. Hystad P. Dzhambov A. M.. (2017). Exploring pathways linking greenspace to health: theoretical and methodological guidance. Environ. Res. 158, 301–317. 10.1016/j.envres.2017.06.028 28672128

[B41] Masselink M. Van Roekel E. Oldehinkel A. J. (2018). Self-esteem in early adolescence as predictor of depressive symptoms in late adolescence and early adulthood: the mediating role of motivational and social factors. J. Youth Adolesc. 47, 932–946. 10.1007/s10964-017-0727-z 28785953 PMC5878202

[B42] McEachan Rosemary R.C. Yang T. C. Roberts H. Pickett K. E. Arseneau-Powell D. Gidlow C. J.. (2018). Availability, use of, and satisfaction with green space, and children's mental wellbeing at age 4 years in a multicultural, deprived, urban area: results from the Born in Bradford cohort study. Lancet Planet. Health 2, e244–e254. 10.1016/S2542-5196(18)30119-0 29880156

[B43] McEachan R. R.C. Prady S. L. Smith G. Fairley L. Cabieses B. Gidlow C.. (2016). The association between green space and depressive symptoms in pregnant women: moderating roles of socioeconomic status and physical activity. J. Epidemiol. Community Health 70, 253–259. 10.1136/jech-2015-205954 26560759 PMC4789818

[B44] Mersky J. P. Topitzes J. Reynolds A. J. (2012). Unsafe at any age: linking childhood and adolescent maltreatment to delinquency and crime. J. Res. Crime Delinq. 49, 295–318. 10.1177/0022427811415284 27867220 PMC5115874

[B45] Mitchell R. Popham F. (2007). Greenspace, urbanity and health: relationships in England. J. Epidemiol. Commun. Health 61, 681–683. 10.1136/jech.2006.053553 17630365 PMC2652991

[B46] Molnar B. E. Gortmaker S. L. Bull F. C. Buka S. L. (2004). Unsafe to play? neighborhood disorder and lack of safety predict reduced physical activity among urban children and adolescents. Am. J. Health Promot. 18, 378–386. 10.4278/0890-1171-18.5.378 15163139

[B47] Mueller M. A. E. Flouri E. Kokosi T. (2019). The role of the physical environment in adolescent mental health. Health Place 58:102153. 10.1016/j.healthplace.2019.102153 31220796

[B48] Office of the Deputy Prime Minister (2001). Generalised Land Use Database Statistics for England. London: ODPM Publications.

[B49] Parker J. S. Benson M. J. (2004). Parent-adolescent relations and adolescent functioning: self-esteem, substance abuse, and delinquency. Adolescence 39, 519–530. 15673227

[B50] Plewis I. (2007). The Millennium Cohort Study: Technical Report on Sampling (4th ed.). London: Centre for Longitudinal Studies, Institute of Education, University of London.

[B51] Quon E. C. McGrath J. J. (2014). Subjective socioeconomic status and adolescent health: a meta-analysis. Health Psychol. 33:433. 10.1037/a0033716 24245837 PMC5756083

[B52] Raghunathan T. Lepkowski J. Van Hoewyk J. Solenberger P. (2001). A multivariate technique for multiply imputing missing values using a sequence of regression models. Surv. Methodol. 27, 85–96.

[B53] Renda J. Vassallo S. Edwards B. (2011). Bullying in early adolescence and its association with anti-social behaviour, criminality and violence 6 and 10 years later. Crim. Behav. Mental Health 21, 117–127. 10.1002/cbm.805 21370297

[B54] Richardson E. A. Mitchell R. (2010). Gender differences in relationships between urban green space and health in the United Kingdom. Soc. Sci. Med. 71, 568–575. 10.1016/j.socscimed.2010.04.015 20621750

[B55] Richardson E. A. Pearce J. Shortt N. K. Mitchell R. (2017). The role of public and private natural space in children's social, emotional and behavioural development in Scotland: a longitudinal study. Environ. Res. 158, 729–736. 10.1016/j.envres.2017.07.038 28750342 PMC5571194

[B56] Ries A. V. Gittelsohn J. Voorhees C. C. Roche K. M. Clifton K. J. Astone N. M. (2008). The environment and urban adolescents' use of recreational facilities for physical activity: a qualitative study. Am. J. Health Promot. 23, 43–50. 10.4278/ajhp.07043042 18785374

[B57] Rivenbark J. Arseneault L. Caspi A. Danese A. Fisher H. L. Moffitt T. E.. (2020). Adolescents' perceptions of family social status correlate with health and life chances: a twin difference longitudinal cohort study. Proc. Natl. Acad. Sci. USA. 117, 23323–23328. 10.1073/pnas.1820845116 31907315 PMC7519389

[B58] Rosenberg M. (1965). Rosenberg: Society and the Adolescent Self-Image. Princeton, NJ: Princeton University Press. 10.1515/9781400876136

[B59] Rubin D. B. (1987). Multiple Imputation for Nonresponse in Surveys. New York, NY: John Wiley. 10.1002/9780470316696

[B60] Smith H. J. Pettigrew T. F. Pippin G. M. Bialosiewicz S. (2012). Relative deprivation: a theoretical and meta-analytic review. Personal. Soc. Psychol. Rev. 16, 203–232. 10.1177/1088868311430825 22194251

[B61] Stouffer S. A. Lumsdaine A. A. Lumsdaine M. H. Williams R. M. Jr. Smith M. B. Janis I. L.. (1949). The American Soldier: Combat and its Aftermath. (Studies in Social Psychology in World War II) (Vol. 2). Princeton: Princeton University press.

[B62] Taylor A. F. Kuo F. E. Sullivan W. C. (2002). Views of nature and self-discipline: evidence from inner city children. J. Environ. Psychol. 22, 49–63. 10.1006/jevp.2001.0241

[B63] Tillmann S. Tobin D. Avison W. Gilliland J. (2018). Mental health benefits of interactions with nature in children and teenagers: a systematic review. J. Epidemiol. Community Health. 72, 958–966. 10.1136/jech-2018-210436 29950520 PMC6161651

[B64] Trzesniewski K. H. Donnellan M. B. Moffitt T. E. Robins R. W. Poulton R. Caspi A. (2006). Low self-esteem during adolescence predicts poor health, criminal behavior, and limited economic prospects during adulthood. Dev. Psychol. 42, 381–390. 10.1037/0012-1649.42.2.381 16569175

[B65] Twohig-Bennett C. Jones A. (2018). The health benefits of the great outdoors: a systematic review and meta-analysis of greenspace exposure and health outcomes. Environ. Res. 166, 628–637. 10.1016/j.envres.2018.06.030 29982151 PMC6562165

[B66] Vanaken G. J. Danckaerts M. (2018). Impact of green space exposure on children's and adolescents' mental health: a systematic review. Int. J. Environ. Res. Public Health 15:2668. 10.3390/ijerph15122668 30486416 PMC6313536

[B67] Weeland J. Laceulle O. M. Nederhof E. Overbeek G. Reijneveld S. A. (2019a). The greener the better? Does neighborhood greenness buffer the effects of stressful life events on externalizing behavior in late adolescence? Health Place 58:102163. 10.1016/j.healthplace.2019.102163 31344526

[B68] Weeland J. Moens M. A. Beute F. Assink M. Staaks J. P. C. Overbeek G. (2019b). A dose of nature: two three-level meta-analyses of the beneficial effects of exposure to nature on Children's self-regulation. J. Environ. Psychol. 65:101326. 10.1016/j.jenvp.2019.101326

[B69] Winkleby M. Cubbin C. Ahn D. (2006). Effect of cross-level interaction between individual and neighborhood socioeconomic status on adult mortality rates. Am. J. Public Health 96, 2145–2153. 10.2105/AJPH.2004.060970 17077398 PMC1698146

[B70] Younan D. Tuvblad C. Li L. Wu J. Lurmann F. Franklin M.. (2016). Environmental determinants of aggression in adolescents: role of urban neighborhood greenspace. J. Am. Acad. Child Adolesc. Psychiatry 55, 591–601. 10.1016/j.jaac.2016.05.002 27343886 PMC4924128

[B71] Zhang Y. Mavoa S. Zhao J. Raphael D. Smith M. (2020). The association between green space and adolescents mental well-being: a systematic review. Int. J. Environ. Res. Public Health 17:6640. 10.3390/ijerph17186640 32932996 PMC7557737

